# Mobilizing Toxins for Cancer Treatment: Historical Perspectives and Current Strategies

**DOI:** 10.3390/toxins12060416

**Published:** 2020-06-23

**Authors:** Jessica Kopenhaver, Robert D. Carlson, Adam E. Snook

**Affiliations:** Department of Pharmacology and Experimental Therapeutics, Thomas Jefferson University, 1020 Locust Street, Philadelphia, PA 19107, USA; Jessica.Kopenhaver@jefferson.edu (J.K.); Robert.Carlson@jefferson.edu (R.D.C.)

The level of complexity in a disease like cancer presents a number of challenges for effective treatment development, which require significant innovation to overcome. Enthusiasm for immunotherapies and other types of biotherapeutics has grown substantially over the past decade, as additional insight into the interplay between tumors and the immune system has allowed for a departure from harsher conventional systemic treatments. However, amidst these impressive advances, these therapies may still fall short for many patients. Faced with this dilemma, more biotherapeutic options continue to be researched as potential primary or adjuvant treatments.

For millennia, poisonous compounds have been used for medicinal purposes such as mild pain relief or numbing during surgery. Even in the modern age, plant and animal toxin-derivatives continue to be widely used as treatments for a variety of ailments. The anticoagulants tyrofabin and hirudin, for example, originate from venom of the African saw-scaled viper and leech secretions, respectively [1]. Even pathogenic bacteria typically considered harmful to healthy tissue may prove to be clinically useful as studies have shown that toxins produced by these organisms can be manipulated to target aberrant cells in a tissue- or cell-specific manner [2,3,4,5].

In this Special Issue, we explore how toxins may be used as powerful treatments against certain cancers. The compiled articles cover how naturally-derived poisons can be utilized for cancer therapy on multiple levels, from interrogating cytotoxic pathways in different cell types, to exploiting toxic derivatives for pain relief in patients suffering from radiation sickness [3,5]. The Special Issue presented this month helps to expound upon this field of research and demonstrate the potential for its clinical applicability.

One of the first uses of toxins as cancer treatments dates back to the early twentieth century, most notably by William Coley, a bone surgeon who discovered that a combination of heat-killed and systemically administered bacteria could shrink osteosarcomas [6,7]. The inception of cancer immunotherapy can arguably be traced back to the innovation of “Coley’s Toxins,” which initiated queries into how a patient’s immune system can be triggered to kill cancer cells [2]. Immunoediting, a prominent idea in the field of immunotherapy [8], asserts that while the immune system is at first able to recognize and kill portions of cancer cell populations, the cancer gradually develops mutations that permit evasion of immune detection, allowing for tumor growth and eventual metastasis [2]. Over the past several decades, clinical strategies to overcome stagnancy in cytotoxic T cell or NK cell responses include utilizing immune checkpoint inhibitors, such as PD-1/PD-L1 or CTLA-4 blockade [9,10,11,12], or direct infusion of cytokines like IFNα [13,14]. Moreover, vaccines against neoantigens [15] and known tumor-associated antigens, could prove useful for patients with genetic predispositions to cancer. Currently, there is a phase II clinical trial investigating the ability of a mucin 1 (MUC1) vaccine to prevent adenoma recurrence in patients at high-risk of colorectal cancer [16]. Oncolytic virotherapy, like the FDA-approved talimogene laherparepvec (“T-VEC”) [17,18], is yet another instance of a therapeutic derived from bioengineering. Remarkably, this kind of virotherapy works to reshape and adapt the tumor microenvironment (TME) to boost immune infiltration.

The mechanism of action for such biotherapeutics must be well understood for effective employment of the treatment, as one study considers. Shiga toxins (Stxs) produced by *Escherichia coli* and *Shigella dysenteriae* 1 pathogenic bacteria bind to the cell surface receptor glycosphingolipid globotriaosylceramide (Gb3) [19] and induce apoptosis by inhibiting protein synthesis [3]. Gb3 is highly upregulated in Burkitt lymphoma (BL) cells [20], and Stx/verotoxins, VT-1 and VT-2, have been used in several preclinical studies, albeit with little success due to abundant cytotoxicity and poor understanding of verotoxin-induced apoptosis [21]. Detailed in one paper, treating BL cells with VT-1/Stx1 consistently induces the endoplasmic reticulum (ER) stress response by activating ER stress sensors, IRE1 and ATF6, as well as increasing expression of the transcription factor C/REB homologous protein (CHOP) that normally signals for programmed cell death. The role of VT-1 in cell death is noted to be cell-specific, and in fact may shield certain tumor cells from death instead of inducing apoptosis. ER stress enhances VT-1-induced apoptosis through CHOP in BL2 cells, but not in Ramos cells [3]. Strikingly, VT-1-induced ER stress triggers ER-phagy that in turn restrains apoptosis in Ramos cells.

*Escherichia coli* protein toxin, cytotoxic necrotizing factor 1 (CNF1), acts as an effective anti-neoplastic in glioma mouse models, reducing tumor volume and increasing survival, all while preserving the functional properties of the surrounding neurons [22,23]. As one paper acknowledges, therapies against glioma cells must be able to cross the blood-brain barrier (BBB), otherwise, treatment would have to be directly injected into the brain [4]. To circumvent invasive cranial injections, CNF1 was reengineered with an N-terminal BBB-crossing tag. Not only does this BBB-CNF1 variant, referred to as the An2-CNF1-H8 variant, show comparable activity to its wild-type (WT) counterpart, but it is also able to be purified in native conditions. This variant also exerts cell growth arrest of U87MG GBM cells in a similar fashion to unmodified WT-CNF1 and upregulates pro-apoptotic protein Bax expression. Experiments performed on endothelial cells demonstrate that the An2-CNF1-H8 variant is able to enter cells and perform its intended functions, as indicated by equivalent actin architecture changes to that of the WT. Intravenous administration of the An2-CNF1-H8 variant upregulates spinophilin in the mouse hippocampus, suggesting BBB bypass. Altogether, these results demonstrate that the An2-CNF1-H8 variant is likely able to cross the BBB to induce cell death in GBM cells [4] and may be translated to future clinical studies.

Similar to clinically-approved CD3-based bispecifics, some immunotoxins utilize antibody-like specificity to recognize tumor antigens, while also possessing a toxic domain that releases a toxin into the target cell following internalization [24,25]. One study explores how immunotoxin efficacy could be improved by modulating the intracellular trafficking of the toxin [26]. The inclusion of a furin cleavage site allows immunoconjugates derived from RNase T1 and the fungal ribotoxin α-sarcin (scFvA33furT1 and IMTXA33furαS, respectively) to be purified with optimized properties for colorectal tumor treatment. It is also noted that the two immunotoxins are trafficked in different pathways after endocytosis. After binding to their target GPA33 on the surface of W1222 colorectal cancer cells, IMTXA33furαS goes through the endosome-Golgi-apparatus network, and scFvA33furT1 appears distributed between the lysosomes and the Golgi-apparatus. The differences in trafficking pathways between the two immunoconjugates align with what is observed from their original constructs [27]. In vitro functional characterization of these variants demonstrates enhanced antitumor efficiency due to increased ability to release their toxic domain into the cytosol, as well as high thermostability and target specificity.

Aside from direct applications as cancer treatments, toxins could be used to mitigate pain directly caused by tumor pressure, or neuropathic pain as a side effect of radiation or surgery in cancer patients [5]. Many clinical studies [28,29,30,31,32,33,34,35,36,37,38] have investigated the use of botulinum neurotoxins (BoNT) as potent systemic analgesics, as these toxins block acetylcholine release from the neuromuscular junction or inhibit neurotransmitters at both peripheral and central sensory levels [39,40,41,42]. Additionally, some sources claim that spiking certain cancer cell lines with BoNT slows growth and mitosis, as well as enhances apoptosis [43]. Studies of pain induced by radiation and/or surgery suggest that the local injection of BoNT improves neuropathic pain and local muscle spasm in the direct vicinity of the site of surgery and/or radiation. However, this type of pain-management therapy requires blinded and placebo-controlled studies to confirm its efficacy [5]. The results from various studies investigating the use of BoNT as an anti-tumor therapeutic also show promise. In several in vivo experiments, direct injection of BoNT into various malignant tumors demonstrated cellular apoptosis and reduction of tumor size [44,45,46]. Adding BoNT (Type A) to a diverse range of cancer cell cultures showed slowed cell growth, as well as induction of apoptosis and reduction of mitotic activity [47,48,49,50,51,52].

Although some cancers have been treated with relative success in the past twenty years, there still remains a paucity of options for patients with difficult to treat, relapsing, or rare cancers. Indeed, cancer is surpassing cardiovascular disease to become the leading cause of death in many populations around the world. This Special Issue presents impactful research that explores the use of toxins as feasible and pertinent cancer therapies which some day may be the solution for so many suffering patients.
